# Is sustainable resource utilisation a relevant concept in Avanersuaq? The walrus case

**DOI:** 10.1007/s13280-018-1032-0

**Published:** 2018-03-07

**Authors:** Astrid Oberborbeck Andersen, Mads Peter Heide-Jørgensen, Janne Flora

**Affiliations:** 1 Department of Anthropology, University of Copenhagen, Øster Farimagsgade 5, 1353 Copenhagen K, Denmark; 2 Department of Learning and Philosophy, The Techno-Anthropology Research Group, Kroghstræde 3, Building 4249, 9220 Aalborg Ø, Denmark; 3 Greenland Institute of Natural Resources, c/o Greenland Representation, Strandgade 91, 2, Postbox 1915, 1016 Copenhagen K, Denmark; 4 Department of Bioscience, Aarhus University, Frederiksborgvej 399, 4000 Roskilde, Denmark

**Keywords:** Atlantic walrus, Greenland, Hunting communities, Management, Sustainability

## Abstract

This article addresses the role of Atlantic walrus (*Odobenus rosmarus rosmarus*) in present-day Avanersuaq from anthropological and biological perspectives, and asks whether or not *sustainable resource utilisation* is a useful concept in northwest Greenland. We describe the relations that unfold around walrus and walrus hunting, in the communities living adjacent to the North Water polynya on the eastern side of Smith Sound. We examine the interplay of walrus population abundance, hunting practices, uses, and formal (governmental) and informal (traditional) ways of regulating the hunt, and we analyse how walruses acquire multiple values as they circulate in different networks. Sustainable resource utilisation, we conclude, is a concept that is relevant in Avanersuaq and beyond, because it works as a biological standard, and hence organises laws, norms, and practices of formal management. Simultaneously, the term is problematic, because it ignores manifold levels of human and societal values connected to walrus.

## Introduction

**Fig. 1:**
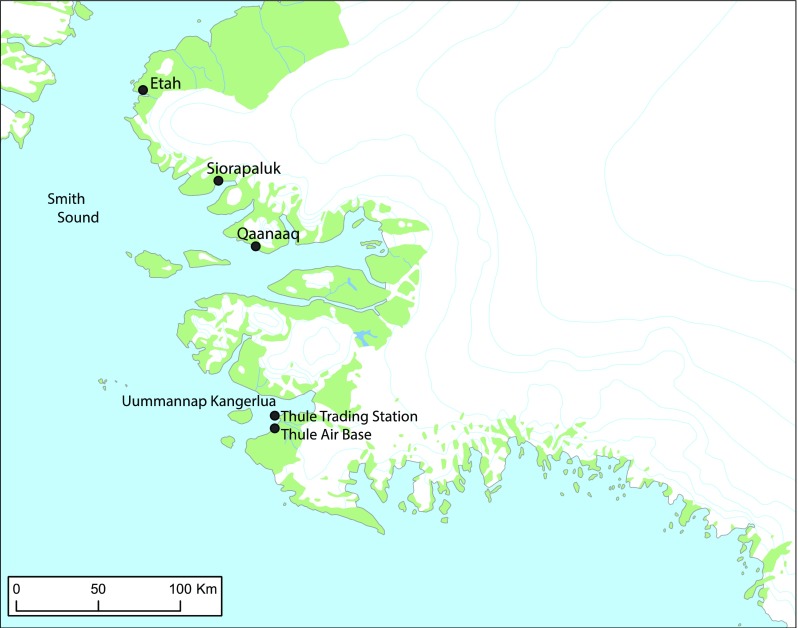
Map of Avanersuaq (Thule area), northwest Greenland

To people in Avanersuaq (Northwest Greenland, Fig. 1), Atlantic walruses (*Odobenus rosmarus rosmarus*) and walrus hunting play important roles in everyday livelihoods. Not only are walruses rich in meat and a good source of protein for humans and dogs alike, the tusks have been used in tools or traded as raw materials, jewellery, or other handicraft, and walrus hunting has been integral in the identity and culture of the people for centuries. Through different historical times, the walrus as animal, meat, and other by-products has acquired differing cultural and economic values to humans inhabiting the High Arctic. This paper explores the question of sustainable resource utilisation. We ask in what ways, and to what extent this concept is relevant in Avanersuaq. We answer this question by exploring the role and importance of walrus in present-day Avanersuaq, and how walruses in Smith Sound are assessed, attain value, and are managed by people living outside Avanersuaq. We integrate findings from biological and anthropological research and trace the walrus through various networks that each produces their own values of walrus. We argue that the walrus becomes a complex and contested socio-biological entity that challenges standard definitions of sustainability and regulation. *Sustainable resource utilisation*, although relevant as a concept, because it affects walrus–human relations, is problematic. The purpose of this article is to show *how*, using a particular species, the walrus, as an example.

Walruses have inhabited Smith Sound and the North Water polynya for millennia. Biologists have disputed whether these walruses belong to one stock, the Baffin Bay—Eastern Canadian Arctic stock (Born et al. 1995), or to three different stocks (Stewart et al. 2014). This paper refers to the walruses occurring in the Smith Sound and adjacent waters as the Baffin Bay stock. Recent biological studies have shown how the North Water is an important habitat for walruses throughout the year (Heide-Jørgensen et al. 2016). These walruses are migratory, and stay on the Greenland side of the North Water from October to May. They cross Smith Sound in June, for summering in shallow fjords and inlets of the eastern coasts of the Canadian High Arctic (Heide-Jørgensen et al. 2017), and are thus absent during the open water season on the Greenland side of Smith Sound. The most important months for walrus hunting among Inughuit (the indigenous people of northwest Greenland) are January–June and October–November. Access to walruses varies in opportunity and difficulty by month of the year. Hunters employ different techniques depending on whether hunting is done on thin new ice in autumn, on shorefast ice in winter or spring, or by boats in open water or semi-open water seasons (see Hastrup et al. 2018a, b). Walruses should be harpooned before they are shot, corresponding to hunting practices prior to the introduction of the rifle, but also to the latest Greenlandic legislation for walrus hunting.^1^ The harpoon is tied to a float (*avataq*) that prevents the animal from sinking, and thus, the loss of the catch. Once caught, the walrus is flensed; the parts are separated according to their intended uses, and shared among the participating hunters according to local customs. The walrus begins to circulate in the hunter-community network where the different parts have their own social significance, and walrus is ascribed value as meat, food, as dog food, as the story of a particular hunt, as trophy, as merchandise. To complete the hunt, some practices are required that connect walruses to a politics-management network, namely reporting the catch to authorities.

**Fig. 2:**
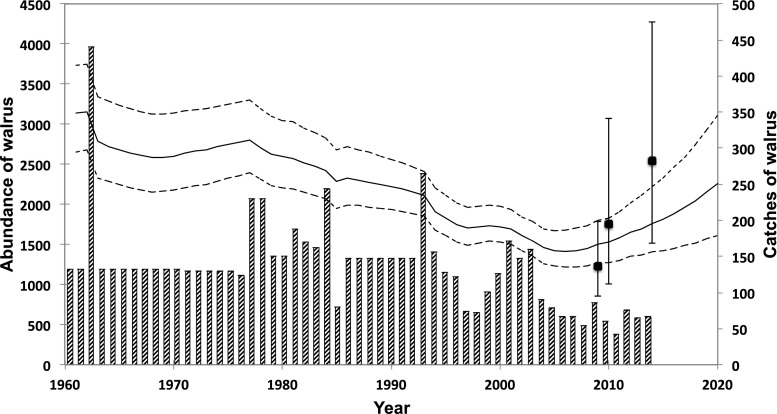
Walrus population trajectory (solid line with dotted lines showing the upper and lower limits of uncertainty) of the Smith Sound walrus population based on the catch history (bars). Catches before 1993 are not well documented and are for most of the years based on extrapolations from occasional reports (see Witting and Born 2014 for catches before 1993). Catches after 1993 are based on Piniarneq through 2006 and thereafter on detailed reporting to the Greenland Government. Catches before 2006 are by occupational and part time hunters, whereas after 2006 only occupational hunters have hunted walruses

For biologists, much effort has gone into estimating current and historical abundance of Atlantic walrus in general, and the Baffin Bay stock in particular (Fig. 2) (Born et al. 1995; Stewart et al. 2014).^2^ To produce abundance estimates, biologists engage with walrus in various ways: deploying satellite transmitters, conducting aerial surveys, collecting samples (for age distribution and reproduction), collecting hunt information (catch and loss), and developing population models. Abundance estimates are important to assess population size and dynamics that inform sustainable levels of removal. In other words, knowing the number of walruses that can be caught while still allowing the population to increase is a primary objective of the data that biologists collect. These biological efforts feed into the political realm of decision-making and management, including determining quotas for walrus hunting. These management decisions then affect human interactions with walrus. Through these political practices, walruses enter the politics-management network in which its values include those as species, stock, and number.

The hunter-community network is different from the knowledge-biology network and the politics-management network, but they are connected and often in conflict. For example, hunters in Avanersuaq question the counts made by biologists, arguing that biologists survey the wrong areas at the wrong time of year, and do not stay long enough to count all animals. Hunters are aware that biologists’ knowledge is the primary knowledge used in wildlife management, but sometimes question its legitimacy. It is not clear to hunters how the number of walruses counted by biologists results in an annual quota of 85 walruses to be caught in 2017, for example. Since the early 1990s, the Greenland government has adopted principles of sustainable use that were developed internationally (Brundtland et al. 1987). With the objective of assessing whether sustainable resource utilisation is a relevant concept useful for wildlife management in Avanersuaq, in this paper, we analyse how sustainable resource utilisation undergoes significant translations when following the walrus around in different networks.

## Analytical framework and core concepts

The accumulation of biological information on walruses in Smith Sound started with field studies in the 1930s (Vibe 1950) and continues today (Born and Kristensen 1981; Stewart et al. 2014; Heide-Jørgensen et al. 2016, 2017). Ethnographic research was part of many early expeditions to the region (Hastrup et al. 2018a); a tradition that continued by Europeans and Americans who colonized and inhabited the area since 1910 at the Thule Trading Station. Specifically for this paper, ethnographic fieldwork based on participant observation, interviews and informal conversations in the case of the anthropologists was carried out in 2015 and 2016. In spring 2015, the anthropologists (authors A.O.A. and J.F.) joined the biologist (author M.P.H-J.) and five hunters on a walrus tagging expedition, as part of a cross-disciplinary effort with the purpose of learning about each other’s ways of knowing (see Andersen et al. 2015). The biologist was interested in where in Canada walruses migrate after leaving Greenland. This is of relevance for the evaluation of the status of the stock, its exposure to hunting in Canada and Greenland, and phenological changes related to new patterns in ice cover. The anthropologists were interested in human practices and uses of walrus and hunting grounds; how Inughuit make sense of and negotiate the shifts and changes in their surrounding land-, ice-, and seascapes.

Our analytical strategy of following the walrus through networks is inspired by Actor-Network Theory (ANT). ANT offers an alternative approach to what in standard social sciences and society is considered ‘the social’. Instead of singling out ‘the social’ as separate from the ‘biological’, ‘economical’, ‘political’ or ‘physical’, ANT traces and accounts for *connections* or *associations* between heterogeneous elements (Latour 1997), human as well as non-human. Inspired by ANT, we trace the walrus and the connections around it in *networks* (Latour 2015). A network is defined as “a string of actions where each participant is treated as a full-blown mediator” (Latour 2015, p. 128). Using network as analytical strategy, the various relations and regimes of knowing, doing and valuing around walrus, become known.

We recognise that *value* is created through particular practices and in relations. Here, value includes monetary and economic value, but also symbolic and cultural worth. *Valuation,* in turn, denotes the practices and processes through which value or values is established, assessed, negotiated, maintained, constructed, or contested (Helgesson and Muniesa 2013). The walrus networks which we explore can be characterised as the hunter-community network, the knowledge-biology network, and the politics-management network.

## Hunter-community network

### A summer walrus hunt

Late, in June 2015, the anthropologists joined two hunters from Qaanaaq on a hunting trip to Inglefield Land. They were in search of walrus and, if the opportunity arose, polar bear too. Both could be found in the northern parts of Smith Sound during this time of year.

We set off from Qaanaaq by dog sledges in two teams. A 20-foot boat, with a 110 hp outboard motor, had already been transported by dog sledge to the first point near the ice edge where it had been used for a narwhal (*Monodon monoceros*) hunt earlier that spring. We left the dogs there, fed, and close to fresh water, before continuing northwards by boat. Nearing Etah in the early morning, two walruses suddenly emerged through the waves close to the boat. Pursuing them for a while, trying to get a good aim, the boat suddenly stopped and turned around. “Our walrus is farther north” said the younger hunter, gesturing northwards with his arm. Landing the right size walrus at the right time, and not just any walrus, is not a trivial matter but requires the kind of knowledge that has been learned and passed through experience for generations. We camped by the ice that still covered the fjord outside Etah and slept through the late morning. By midday, we found a lookout post and scouted for walrus. There were none and we continued northwards. Little islands off the coast, here, and elsewhere in the area have been named after walrus body parts; for example, Taliilaq is an island shaped like a walrus flipper.

Walrus hunting is an activity through which cultural values are performed and reproduced. Knowing where the walruses can be found, travelling to reach them, and recognising walrus of different shapes; as living animals (potential prey), and as places that are named with walrus body parts, are activities loaded with cultural significance and expertise. Identifying the right walrus as catch, catching it, transforming the entire animal into parts by preparing them to become different foods and materials that will circulate in society, are all cultural ways of producing walrus values. Once the walrus has been transported to the settlement or town, some of its parts can even be transformed into monetary value.

Walrus meat is recognised as the best dog food followed by bearded seal meat. Walrus meat keeps the dogs’ paws and furs in good condition. For humans too, walrus is food, but dogs are the higher priority; therefore, for human consumption, walrus is becoming a rare delicacy enjoyed mainly at celebrations. Hunting restrictions imposed by municipal wildlife management limit the number of walruses that a hunter can catch on a given trip. For each hunting trip, a hunter must acquire a license to catch walrus. The municipal council in Qaanaaq issues numbered licenses, and only hunters with a valid vocational hunting certificate can acquire a license. For one hunting trip, the maximum number of walruses that can be caught in Qaanaaq is two. For each license, hunters must fill out forms and hand these to the municipal council after they have caught a walrus.^3^ The format of this document is the same for all municipalities and walrus stocks in Greenland. It is formulated by the Ministry of Fisheries and Hunting, and agreed upon by the Greenlandic Parliament (*Inatsisartut*) as part of the general legislation for walrus hunting.

**Fig. 3:**
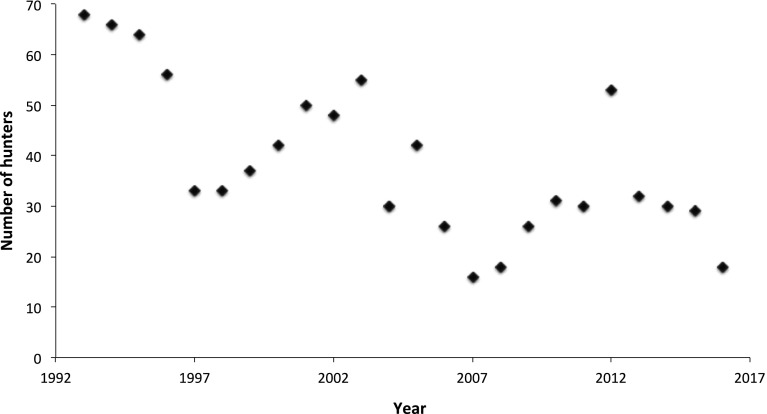
Number of walrus hunters, only occupational hunters, in Avanersuaq from 1993 to 2016 (data from Piniarneq)

Travel by boat to the northern hunting grounds is costly and possible only by the hunter-maintained depots of petrol along the coast. The success of walrus hunting is contingent on careful logistic planning. Returning without walrus meat is not an option. Even so, the two walruses the hunters were allowed to bring home from this hunt are not enough to feed two teams of dogs for the months that are ‘closed’ for walrus hunting, beginning only a few days later on 1st July. One big walrus, we were told, will feed a team of 16 dogs for a little less than a month, and hence, if dogs were to eat walrus meat only, each hunter would need 12 walruses each year. With around 60 full-time hunters in Avanersuaq in 2015, feeding dog teams with only walrus would require a quota of 720. Needles to say, 85 are not enough. Dogs eat all kinds of meat, blubber, fish, and kitchen leftovers, and dog food can also be bought in the store. However, the number of walruses caught has been below 100 since 2004, suggesting that there are fewer occupational hunters and, subsequently, fewer dog teams to feed. The number of occupational walrus hunters in Avanersuaq, and hence the amount of dog teams, tends to fluctuate (Fig. 3).

“Our walrus” did, indeed, turn out to be farther north. We spent the following day boating to the shores near the northern limits of the North Water. We went ashore and the hunters scouted northwards towards the pack ice, hoping to see walrus. A polar bear (*Ursus maritimus*) walked in the far distance, and soon after the hunters spotted a herd of walrus on the pack ice. We returned to the boat and approached the walruses quietly. As we neared the pack ice, the younger hunter grabbed his harpoon and rifle. He disembarked and quickly jumped onto the ice from where he harpooned a male walrus and quickly delivered a precise shot. Now, began the arduous task of hauling the walrus onto land, flensing, and packing the boat. The younger hunter, who owned the boat and had landed the walrus, was awarded the head, tusks, and penile bone. The rest of the midsize walrus was shared, lengthways, in accordance with old customs. The stomach contents, mainly mussels, were studied carefully, tasted, and gathered for eating later. The older hunter noted that he could only see two of the three kinds of mussels walrus usually feed on. Flippers, ribs, back, all stacked into two neat piles of meat. The hunters worked quickly against the turn of the tide, and before long the walrus was transformed into food; some for dogs, and some for humans. The human food for delicacies required different kinds of preparation. We boiled the heart for dinner, eaten shortly before bedtime, which after a long day was early in the morning.

**Fig. 4:**
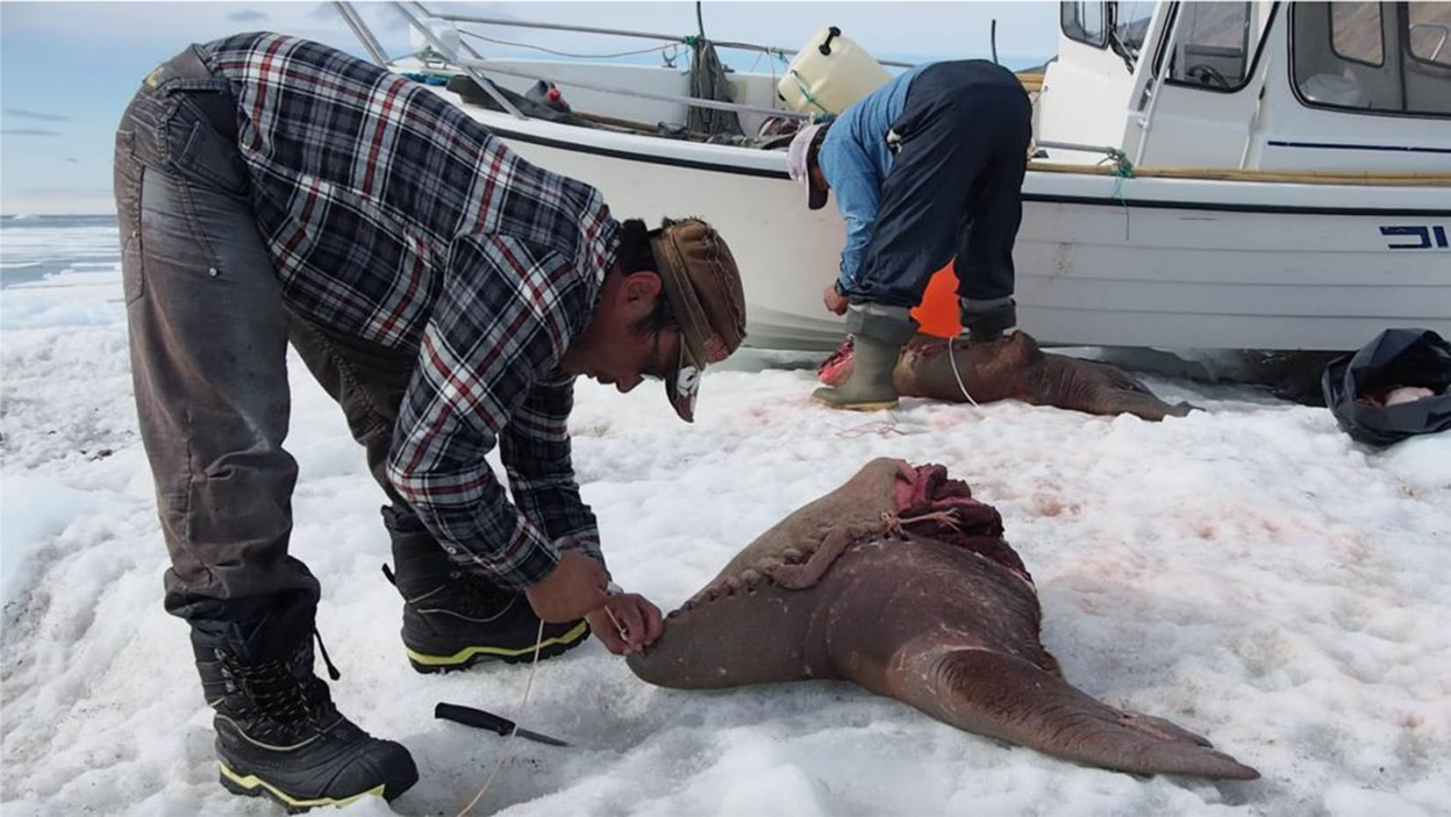
Hunters Mads Ole Kristiansen (front) and Kristian Eipe (back) making their ungerlaaq. Photo: Astrid Oberborbeck Andersen

To lighten the load of our return journey, the hunters sewed up two of the flippers, one foreflipper, and one hinterflipper, into neat parcels (called *ungerlaaq*) to be preserved under a heap of rocks on the island until they would return later in summer to pick them up. Upon returning to the hunting cabin where we had set out by boats a few days earlier, the two hunters repeated the process with the remaining flippers. This time adding liver and kidneys, but using the same technique of stitching together the flippers and burying under rocks, where it ferments. Traditionally, *ungerlaaq* was one of several ways people preserved foods for the months of winter where hunting was difficult. Today *ungerlaaq* is enjoyed mainly at times of celebration. As they placed their stitches carefully, the hunters speculated when they might be ready to eat. The younger hunter hoped his would be eaten at his mother’s birthday in January. As it turned out, due to the warmer summer, it was ready for his daughter’s first day at school a couple of months later (Fig. 4).

The walrus takes different shapes and forms through its circulation, initially as desired and imagined, and eventually as concrete potential food with different tastes, textures, and purposes. As the walrus is flensed and transformed into different kinds of food-in-the-making, we note how it already begins to circulate in social networks and occasions that each in their own way produce value around the walrus as animal and walrus as food. Simultaneously, as the document of catch is filled out and handed into the municipal authorities, the walrus begins to circulate in the bureaucratic network of wildlife management.

### The economic significance of walrus

Present-day Inughuit hunting livelihoods are a mix of cash and subsistence economies. Although far away hunting grounds can be reached much faster nowadays with motorised boats, travelling is a costly affair. Hunters need cash to sustain their hunting activities, and are sustained by subsidies on fishing equipment such as boats and outboard motors, and earnings by fishing for halibut (*Reinhardtius hippoglossoides*) in winter and spring, or selling *mattak* from narwhval and beluga (*Delphinapterus leucas*) to Royal Greenland that supplies the market in the south (see Flora et al. 2018).

This has not always been so. Before the market for seal skins collapsed in the 1980s and 1990s, through systematic campaigns against seal hunting and changes in the fashion industry, Inuit hunters in Greenland and Canada earned an income by trading seal skin. In the 1960s, for instance, a good hunter in Greenland could earn an income resembling that of a schoolteacher in Denmark by the trade of seal skins alone (Hastrup et al. 2018b). As a consequence of intense campaigning by international organizations, the seal fur market collapsed almost overnight. Although seal hunting in Greenland has always been evaluated as sustainable, seal skin products became unfashionable and unethical, and as a result, the price that hunters in Greenland and Arctic Canada could obtain for a seal skin plummeted (e.g., Wenzel 1991). Though hunters still sell skins today, much of their effort is directed towards other species of sea mammals that can generate cash income; in Avanersuaq, these are narwhals, Greenland halibut (*Reinhardtius hippoglossoides*), and tourists, and to some extent walruses and belugas.

In comparison to seals, walruses and narwhals cannot sustain the same hunting pressure, because their populations are smaller and because they are slow to reproduce. Occasionally, walrus meat is sold locally to families craving for the taste of this meat. It is mostly the women’s responsibility to distribute meat and shares to extended networks in the town. The Women’s Association and the hunters’ association in Qaanaaq have agreed to standard prices for meat to avoid haggling. Walrus meat without bone costs DKK 50 (USD 7,20) per kg,^4^ the walrus heart DKK 75 (USD 10,75) per kg. Mussels from the walrus stomach cost DKK 100 (USD 14,33) per kg, and the *ungerlaaq* is DKK 60 (USD 8,60) per kg. Walrus tusk ivory is used sometimes to carve hunting tools, but is also sold locally to craftsmen who carve figurines or jewellery. These by-products, as well as the cranium with tusks, are sold to tourists or other visitors. Prior to 2007, when the Greenland Government first introduced annual quotas for walrus in the eastern part of Smith Sound, hunters could catch the amount of walruses that they needed for their families and dog teams to subsist. In addition, trade in tusks can provide a good income, although trade in some years has been restricted to the domestic market. International trade of walrus ivory was restricted due to regulations by the European Union in 2008 and CITES (Convention on International Trade in Endangered Species), which Greenland joined in 1992. Although quotas were not introduced until 2007, old unwritten rules, and local decrees from 1929 have dictated the hunters not to overhunt walruses or other animals. In addition, although local exploitation of walrus decreased already before the introduction of quotas in 2007 (Fig. 2), hunters lament the restrictions put on walrus hunting via quotas (Table 1), as they can no longer catch the amount necessary for their dogs to become strong. Since the low level of catches of walrus in 2004, and the introduction of quotas, the number of occupational walrus hunters has increased (Fig. 3), suggesting that the amount of dogs and demand for walrus meat has gone up. “The law says dogs and children should not starve. It hurts that we are not allowed to catch what we need,” one hunter claimed during a public meeting in Nuuk in 2017, about current hunting routes and catches in Avanersuaq. In addition, “who needs many dogs these days, anyway” he continued, after stating that he had reduced his dog team during summer.^5^ Hunters experience a loss of the right to hunt the walrus that they need.

**Table 1:** Development of quotas for walrus hunting on the Greenland side of Smith Sound. The advice is given by The North Atlantic Marine Mammals Commission (NAMMCO)

Year	Quota	Advice for Greenland
2007	90	
2008	80	
2009	75	
2010	64	68
2011	64	68
2012	64	68
2013	74	89
2014	86	89
2015	86	89
2016	86	85
2017		85
2018		85
2019		85
2020		85

When it comes to converting walrus parts into monetary value, the hunter-community network extends beyond Avanersuaq, to those interested in purchasing walrus by-products, and entities (CITES, IUCN, EU) concerned about protecting global biodiversity and wildlife. Among these entities, walruses are valued as a potentially endangered species that must be protected by international conventions and regulations. However, the primary concern to hunters and people in Avanersuaq is food security for families and dog teams, which is not only a concern about calories but feeds into concern about the continuation of hunting livelihoods. Recently, the hunts of all three stocks of walrus in Greenland were found to be sustainable, and awarded a positive CITES export declaration (i.e., a ‘non-detrimental finding’), which means that export of walrus products from Greenland is conducted without harming the stocks of walruses.

## Knowledge-biology network

### Knowing and assessing walrus abundance

Concerns about the abundance of a species are central to the concept of sustainable resource utilisation, and these concerns are not new. Peter Freuchen, explorer and co-founder of the Thule Trading Station, was the first Westerner to produce a systematic written account of the appearance and migration routes of walrus in West Greenland. In his text, Freuchen (1921) describes the walrus population in West and Northwest Greenland as one population that every year migrates in an anti-clockwise circular motion in Baffin Bay; northbound along the coast and ice edge of West Greenland during months of spring, and southbound along the Canadian coast in summer and autumn (Freuchen 1921). Although his observations of the population have since been proven to be incorrect (Born et al. 1993), the description is relevant, because it provides a picture of some hunting grounds, described through the seasonal appearance of walrus, and how walrus were harvested in this area, in the beginning of the twentieth century:

In spring time, hunting on ice at Haklyut Island, which is never connected to the other two islands (Northhumber Island and Herbert Island) by ice, is big. Immeasurable amounts of walrus are caught here, and still, there are enough, still new animals arrive, but it seems as if they are no longer in the haste they used to. They have now nearly reached the end of their journey. Yet, still some way, they have to fair before reaching the place that among polar eskimos is the most famous of all the walrus hunting sites. That is Pituravik and its closest surroundings, where the sea in summer and winter sometimes boils from the snorting of the animals. (Freuchen 1921, p. 243; translated by authors)

Freuchen’s descriptions are an attempt to make an account of walrus abundance and migration in the region. They are based on his own observations from travels through the landscape as hunter and as explorer, supplemented by other hunters’ accounts. Notably, he assesses abundance—“enough”—not in relation to a stock status, but in relation to what is necessary for hunting and human survival. Already at this point, Freuchen assessed the prevalence to be less than in earlier times; and today, walruses are no longer found on the Greenlandic side of Smith Sound in August, a month otherwise described by Freuchen as rich in walrus, leading Witting and Born (2014) to suggest that the total amount of the Smith Sound stock has declined significantly since 1921. Freuchen’s account predates formal management policies based on principles of “sustainable resource utilisation”. However, it points to a close relation crucial to ideas of sustainable resource utilisation between assessing abundance of a given resource, and specific human uses of this resource.

### Biological walrus studies

In late May 2015, we went on a walrus tagging mission, along with five hunters from Qaanaaq. The mission was part of a longer series of biological surveys of walrus carried out in Smith Sound, where walruses were tagged with satellite transmitters. One of the purposes was to learn more about where the walruses roam during summer after they abandon the eastern part of the North Water. The biologists work in close collaboration with local hunters who had two roles. One role was to serve as logistical operators using dog teams and boats, in a world they know better than anyone else. The other role was to attach the satellite transmitters into the skin and blubber of the walrus, using their skills of approaching and harpooning walruses. The five hunters started to prepare a couple of weeks before the biologist arrived. Boats had to be lifted onto specially crafted sledges and taken to the edge of the sea ice together with plenty of fuel. This kind of collaboration requires mutual trust and precise coordination of activities, and on occasions like this, the walrus networks of hunters and biologists come together. Activities around the walrus overlap and merge, although in somewhat different ways.

**Fig. 5:**
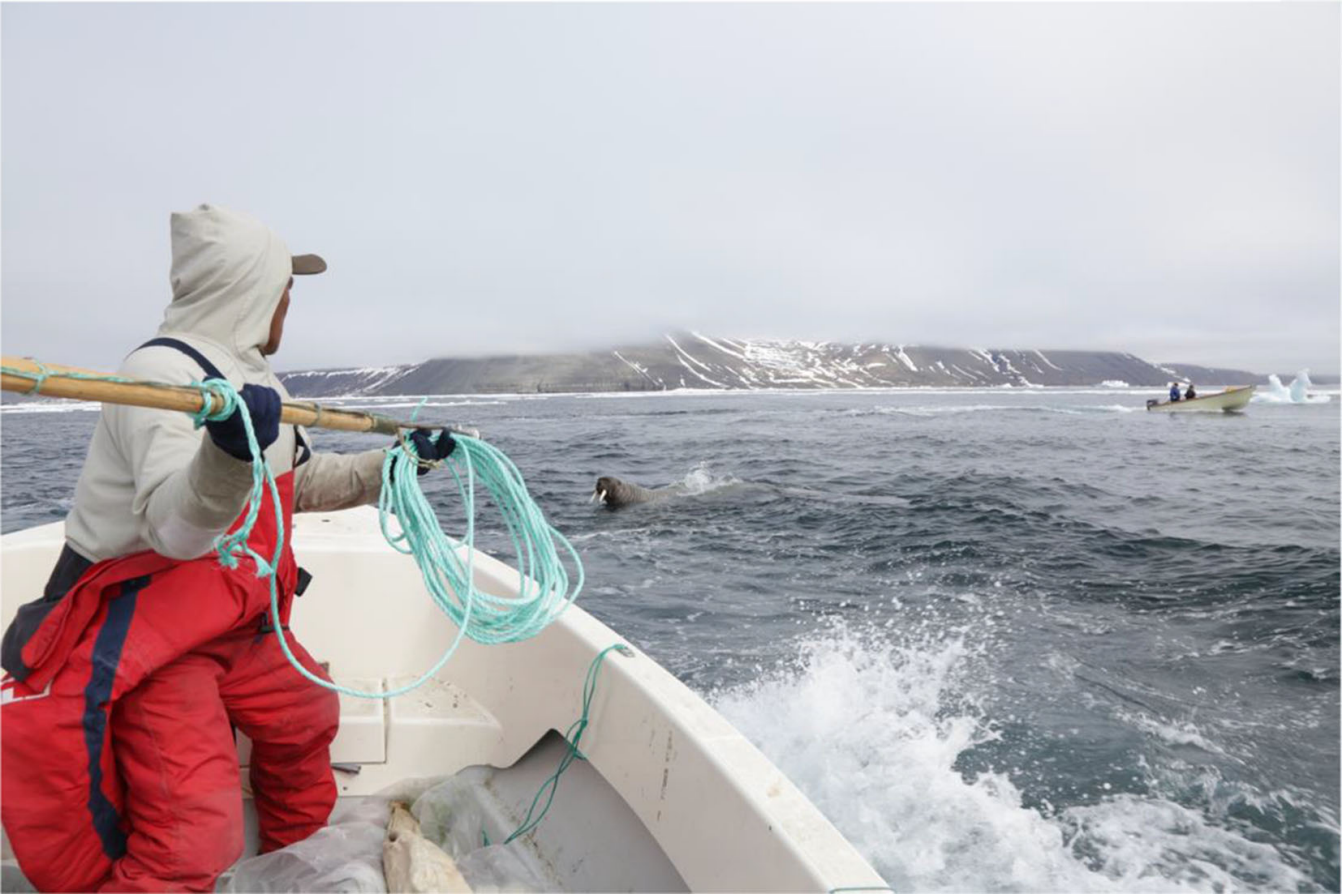
Tagging walruses in water. Satellite transmitter mounted on hunter’s harpoon. Photo: Mads Peter Heide-Jørgensen

Based on the conditions of the sea ice, and on hunters’ considerations about where large aggregations of walrus reliably could be found, the location of the tagging was decided between biologist and hunters, once the biologist had arrived. We departed in boats from the ice edge, southwards into Uummannap Kangerlua (Wolstenholme Fjord) close to the Thule Air Base, where the previous generation of Inughuit was evicted and forced to relocate to Qaanaaq in 1953. Before then, this area was a hunting ground known for its abundance of walruses in June–August; beginning when the ice broke and the mussels were accessible to the walruses in the shallow waters (Freuchen 1921, pp. 241–242). These days, it is a place where hunters pass through to sell polar bear fur, walrus, and narwhal tusks to the American and Danish employees on the Air Base and occasionally to hunt walrus. Walrus counts by airplane in April 2014 revealed groups of walrus in the inner parts of the fjord (Heide-Jørgensen et al. 2016); an area that until recently was inaccessible to walrus due to the sea ice that would remain far into June. In late May 2015, within 8 h, 19 out of 21 transmitters were placed on walruses in this fjord. The hunters moved quickly, the boats at times driving together. Some walrus were tagged while sleeping on ice floes; others were tagged in water (Fig. 5).

Although the tagging took 8 h, the mission lasted 5 days, delayed by weather and ice conditions. During these 5 days, the walrus network of hunters overlapped with that of the biologist, for instance when a walrus reared his head immediately next to one of the boats, one hunter laughed and shouted: “You’re tasty!” Although hunters were on a scientific mission and not on a hunting trip, they still saw meat and taste in potential during the task of tagging. This shows how different kinds of walruses, and different networks existed in the same time, space, with differing human perspectives. The hunters earned a wage for tagging, which was an unusual kind of engagement with walrus; an interesting collaboration with researchers, and an exercise in hunting. For the hunters, the walrus tagging enters their chain of walrus actions as a kind of wage labour through which they accumulate knowledge about their hunting ground, and practice hunting techniques. However, the meat normally associated with a long hunting trip was not part of the arrangement. For the biologist, the tagging is only the beginning of the production of new knowledge about walrus migration patterns, diving, and foraging habits. After the tagging, the signals of the transmitters are captured by ARGOS, the worldwide location, and data collection system, and sent to a platform that can be accessed on the internet. Through these practices, the walrus enters the knowledge-biology network as a scientific object.

### Biology-based and political-driven population modelling and projection

Biological engagement with walruses in Smith Sound began in the first part of the twentieth century when Danish zoologist Christian Vibe initiated studies of wildlife in Avanersuaq, and described the behavior of walrus and other sea mammals (Vibe 1946). Since then, several studies, linked to different knowledge interests, have been carried out, and research methods have evolved over the years. Extensive collections of samples from the walruses were conducted in the 1970s and 1990s with the purpose of determining the age structure and productivity (age at sexual maturity, fertility, and pregnancy rate) of walruses in Smith Sound (Born 2001, 2003). Samples for genetic studies have also been processed to examine the identity and discreteness of the walruses in Smith Sound and adjacent waters (Andersen et al. 1998; Andersen and Born 2000; Born et al. 2001). Biological studies of the walrus population in Smith Sound are designed and conducted independent of management, but increasingly with the objective of producing scientific advice for monitoring and management.

For walrus (and other marine mammals in Smith Sound), the information needed for managing the stocks at maximum sustainable yield levels (MSYL) is not available. Surveys of walrus abundance are only available for the past decade and a long-term time series of abundance estimates that can illustrate the relation between hunting and abundance are missing. Information on reproduction and annual survival rates of different age classes of walrus is difficult to obtain and has only sporadically been collected but is not considered critical for the population modelling. Instead, simpler population models that assess the risk of future catches on the estimated abundance are used for guiding the advice on catch levels (Fig. 1). The models utilize a history of catches in Northern Canada and Northwest Greenland corrected for underreporting and losses, together with recent surveys of the abundance of the shared Greenland-Canada stock of walrus in Smith Sound and adjacent areas. The model includes assumptions about the range of annual survival (0.95–0.99), first-year survival (0.5–0.9), age at first reproduction (5–9 yrs), and birth rate (0.35–0.65) based on realistic values from other stocks (see Witting and Born 2014 for details on the model).

Five-year stock projections are then used to assess the risk of decline under different catches. The target is to maintain the stock at present level or to allow it to increase. For management purposes, it has been accepted by the Greenland Government that future catches should with at least 70% probability allow the stock to increase when evaluated over a 5-year period. This should eliminate the risk of further decline and at the same time ensure that the stock can maintain a large surplus production in the future.

This type of population modelling relies on frequent and relatively precise surveys of the abundance of walrus that can be used for adjusting possible mistakes in the five-year population predictions. It also depends on reliable catch statistics from both Canada and Greenland for at least the past 50 years, and estimates of walrus that are struck and lost and, therefore, not reported. Thus, frequent abundance estimates become increasingly important if accuracy of the catch reporting deteriorates.

The settlements of Northwest Greenland were not included in the old Hunters List of Game (HLG) that the Danish authorities implemented in the 1860s in West Greenland, and it was not until the 1950s that catches from Northwest Greenland occasionally were reported through the HLG. Large catches of 440 walruses were reported in 1962 and the average for the other years in the 1960s was 132 walruses (Witting and Born 2014). Proper statistics on catches are not available before 1993 when *Piniarneq*^6^ was introduced. During the 1970s and 1980s, estimates of catch levels are occasionally available from observations by visiting scientists and from the HLG. For missing years, averages of the previous years have been assumed to apply.

Loss rates during the hunt of walrus are even less well documented, and as it has changed with the introduction of motorboats. Loss rates were considered minimal (~ 5%) before 1960 and the introduction of motorized vessels where walrus were always harpooned before being shot (Witting and Born 2014). With the increased use of motorboats in the 1960s, losses were assumed to increase gradually and losses of 30% were assumed for catches after 1970 (Born and Kristensen 1981). Although the estimates of loss are rough, it is safe to assume some losses, especially for catches of females with calves where calves are often lost without being reported.

Walrus are widely dispersed in Smith Sound and adjacent areas during all times of year making it unlikely that a single survey can capture the entire population in a short time frame. Instead, surveys have focused on providing the largest possible estimates in areas where walruses are known to aggregate during certain seasons (Heide-Jørgensen et al. 2016). Estimates of carrying capacity based on the extension of shallow water (< 50 m) feeding grounds for walrus make it however unlikely that the current walrus population in Smith Sound and adjacent areas can exceed ~ 5.000 animals (Garde et al. 2018). This sets an upper limit for both current and historical abundance estimates, and hence also for sustainable use.

Despite the uncertainties involved in reconstructing the catch history including extrapolations from sporadic observations and assumptions about loss rates, it is still important for modelling the population trajectory, as periods with few or zero catches would result in low estimates of the productivity in the population and thereby similarly low predictions for future sustainable catch levels. In practice, all variables (catch history, abundance estimates, survival, and reproduction) are, however, treated as uncertain predictors of the population development and the safe space for future catches is estimated statistically. Through the biological approach, the walrus emerges as a being that can and must be surveyed, estimated, sampled, and modelled, it makes a stock or a population, of a species that can be compared to other species through these measures.

## Politics-management network

It is generally accepted that the introduction of large boats with powerful engines, which most hunters use today, has caused a major change in hunting patterns and effort, both by expanding the operational range of the hunters, but also by allowing larger catches to be brought back much faster than what was possible just 1 decade ago, increasing the risk of population depletion. Likewise, it is widely accepted that these changes in use demand regulation on the harvest of animals (Born et al. 2017). What continues to be debated is *how* management is carried out most effectively.

The hunt for walrus in the eastern part of Smith Sound (the Greenlandic side) has been formally regulated since 1949, when the season for walrus hunting was first limited, and walruses all around Greenland could only be hunted from January 1st to May 19th. This restriction was triggered by changes in hunting practices and observations of particularly high catches of walrus in West Greenland (Wiig et al. 2014). In 1992, The North Atlantic Marine Mammals Commission (NAMMCO) was formed, and the Greenland government signed the NAMMCO Agreement on Cooperation in Research, Conservation and Management of Marine Mammals in the North Atlantic. Upon signing this agreement, the Greenland Government, with other North Atlantic governments, was explicitly committed to the principles of sustainable resource utilisation. The agreement builds on “the general principles of conservation and sustainable use of natural resources as reflected in the report of the World Commission on Environment and Development” (NAMMCO 1992:1), and hence, the link to international organizations such as the Brundtland report (1987) through which sustainability is traceable.

In 2006, the Greenland government introduced the first quotas on walrus hunting. However, due to uncertainty about the abundance of Atlantic walruses, the enforcement of these quotas was delayed until 2007 (Wiig et al. 2014). Since then, the kind of information biologists can provide, in terms of abundance (stock estimates), migration patterns, and carrying capacity, has been increasingly crucial to support the management of exploitation of walrus. Currently, the politics-management network cannot function without biology. Regulation of quotas in Greenland is based on advice on ‘sustainable catch levels’ provided by the North Atlantic Marine Mammal Commission (NAMMCO). The recommended catch levels for walrus (and other species under quota regulations) are partly based on the paradigm that exploited populations (stocks) have maximum sustainable yield levels (MSYL) at population sizes where the surplus production is at an optimum, with the lowest mortalities and the highest reproductive rates. The basic idea has been developed within fishery assessments for half a century and it predicts that there is a link between stock abundance and reproduction on one side and mortality in the form of natural and hunting or fishing mortality on the other side. There is both empirical and theoretical support for the idea that certain levels of exploitation will maintain stocks at levels where the largest yields potentially can be taken (Beverton and Holt 1957). It is well recognised, however, that attaining and maintaining stocks at MSYL are not simple and in many cases cannot be achieved possibly in part due to a highly variable marine environment and resulting in a fluctuating carrying capacity. In order for utilisation to become sustainable, several factors and forms of information must be in place.

After the first introduction in 2007, the quota has gradually been reduced from 90 in 2007 to 64 in 2010 when formal advice based on a biological assessment was provided by NAMMCO. This quota is only for the Greenlandic side of Smith Sound, since no co-management agreement of the stock has been made between Greenland and Canada. The biological advice by NAMMCO was updated in 2012 and 2015 following biological surveys of the abundance of walrus in Smith Sound, carried out in 2009/2010 and 2014 (see Fig. 2).

The Greenland Government’s efforts at managing the exploitation of living resources are tied to multinational (NAMMCO) and international (CITES) ideas of sustainable use of wildlife. As we have seen, biological information provides the foundation for setting and using a quota-regulation apparatus by illustrating scenarios for population growth and assessing biological sustainability under certain management regimes; that is, assessing how many walruses can be caught from each population without preventing the population from growing. The other part of the quota-regulation is the political process with public hearings and consultations with hunters and their organisations. All proposed legislation for walrus hunting and protection enters public hearing: the national association of hunters and fishermen (KNAPK), the association of Greenlandic municipalities (KANUKOKA), the municipalities that are involved, the Greenland Institute of Natural Resources, among others. Hunters in Avanersuaq are organised in a local branch of KNAPK, and are able to state opinions through those organisations alone. This formal road to influence is rather complex, and in practice, the channels through which hunters in Avanersuaq are actually able to influence hunting regulations are variable and ad hoc, depending on personal connections and negotiation skills. *Sustainable catch levels* is not a term or a concept that hunters use in such negotiations; rather, they talk of regulation in more implicit terms, to which we now turn.

## Sustainability: Concepts of resources as renewable and exhaustable

The concept that marine resources are exhaustible comes from more than 100 years of biological research, empirical data, and from fish stocks subject to commercial harvest, but also from local observations of bird resources being vulnerable to excessive exploitation (e.g., Faroe Isles). Biologists and politicians have examples of populations that were overexploited and so know that it can happen. It is not, however, a concept that is integral to hunting culture in Northwest Greenland, because there are few historical examples of depleted populations (caribou and muskoxen), and the general experience is that animals cannot disappear, and there are enough animals to take what is needed. Natural resource managers have found that it is difficult to provide information about the scientific background for the quotas and the necessity of having restrictions on the catch levels in a way that the hunters in Greenland can fully understand. Ideas about conservation, sustainability, and resources from nature being exhaustible mainly have their origin in seventeenth century European worldviews, and come from sedentary forms of producing and harvesting resources, such as forestry and fisheries (Arler 2015). The term “resources” originates in an economic view of what humans can take from nature to produce other goods. These ideas are radically different from the traditional Inuit and Inughuit ways of relating to and using land, ice, sea, and game. Ethnographic literature from across the Arctic is rich in descriptions of animals and humans considered sentient beings, because animals and humans alike have consciousness-giving souls (Rasmussen 1929; Fienup-Riordan 2000; Petersen and Lynge 2004). Although sharing a somewhat similar kind of personhood (Mauss 1985 [1938]), humans and animals are positioned differently in the world, as hunter and prey, respectively. The hunter–prey relation is rather general, but changes regionally, and according to the specific animal, its habitat and the way it is hunted. Although these ideas vary around the Arctic, generally speaking, central to Inuit concepts of the human (Nuttall 1994; Flora 2015) and non-human animal soul is the notion of the soul returning to a new body after death as a kind of reincarnation. The continuity of the animal populations is ensured (Fienup-Riordan 2000; Obeyesekere 2002). Guemple (1994) describes how, among Qiqiqtamiut in southeastern Hudson Bay, animals and humans have a limited supply of souls that circulate in a closed circle of birth, death, and rebirth. Guemple characterises the cycle of souls as “a zero-sum game: no new ‘players’ can enter the system, none can permanently depart. They can only be recycled or displaced to some other location” (Guemple 1994, p. 120). In such a worldview, animals are finite and renewable at the same time.^7^ In these notions of life and relations to the living beings in the landscape, living resources are not immediately understood as exhaustible among present-day Inuit and Inughuit hunters (Rosing 1998). This does not mean that hunting has not been self-regulated before states imposed restrictions and quotas on hunting. There are many examples of how hunting has been organised by ritualised practices and rules, as well as principles of not catching more game than needed (Rosing 1998; Laugrand and Oosten 2015). One principle that has traditionally guided hunting and trapping in the Arctic is that animals offer themselves to the hunter. Catching an animal then becomes a deed of respect towards the animal’s will, and the hunter responding to its request. If not respected, animals could potentially revenge themselves on the hunters by not showing themselves again (Rosing 1998; Laugrand and Oosten 2015, pp. 64–65).

In this way, many unwritten rules and traditions guide hunting practices, and the relation between humans, animals, and hunting grounds. Hunters in Avanersuaq respect these rules, claiming that they protect their hunting area by conserving old hunting practices. Young hunters are taught to use kayak and harpoon when hunting narwhal, and this is considered the best way to protect and conserve the animals and the hunting area. The concept that the hunting area is also an entity to be conserved speaks to the coexistence of animals and the human utilisation of these animals. “We cannot deplete the animals”, we heard a hunter state at a public meeting, “we have been taught to only catch what we need.” Another hunter stated that nature itself, including climate change, protects the walrus from overhunting, by restricting human access to the animals through the seasons, and by providing less sea ice on which to go hunting.

Many hunters in Avanersuaq emphasise “traditional hunting practices” when explaining their relation to the landscape and hunting areas, and when arguing against quotas on walrus, narwhal, polar bears, and belugas. However, they also refer to the early attempts of formalising local hunting restrictions. The first of these is the Thule law (*Kap York Stationen Thules Love af 7. juni 1929*), introduced in 1929 as a legal device used in the administration and daily governance of the Thule trading station (*Kap York Stationen Thule*) founded by Knud Rasmussen and Peter Freuchen in 1910. Although the Thule law was officially replaced in 1963 when the Thule area was embraced by Danish–Greenlandic law (Hastrup 2015), some hunters still refer to it when explaining the old rules they follow to protect the hunting areas. Another mode of restricting hunt is a set of rules introduced by the local hunters’ association in the 1950s. According to a board member of the hunters’ association today, the hunting grounds around Qaanaaq were divided into three zones, each with restrictions on hunting corresponding to the wildlife activities in it. Zone 3, for example, is the innermost part of Inglefield Bredning, where narwhals occur in thousands every summer. In this area, no traffic in or hunting from motorised boats is allowed.

### The Thule law

The Thule law was formulated and put in force by Knud Rasmussen and a so-called hunters’ council (*Fangerråd*) in 1929 (Harhoff 2000). At that time, Rasmussen was owner and governor of the trading station. The hunters’ council was established in 1927 to make administrative decisions and to act as a participatory organisation and authority in the community around the trading station. Among the first decisions made by the council were rules set to protect and conserve foxes and eider ducks. The council consisted of six “good and knowledgeable men” (Kap York Stationen Thules Love: A.), of which three were Danes working in the administration of the trading station, and three were Inughuit representing the three parts of the Thule District: North, Thule, and South. Together, these men represented the ‘tribe’ as a whole, and functioned as legislative, executive and judicial power (Harhoff 2000) at the station and in the Thule District. Knud Rasmussen, as owner of the station, however, had the authority to make decisions beyond the law. The law looked to promote the wellbeing of individuals of the tribe and of the society as a whole, and although the Thule District was integrated into Danish Greenland in 1937, and the law was never juridically recognised by the Danish state, it had status as a set of provisions, and was enforced, practiced, and the text was even modified by Danish ministerial authorities until 1963.

Occasionally, the Thule law’s historical context as well as parts of its juridical content and status is referred to in present-day Greenland, by hunters in the Thule area, and politicians in Nuuk alike. In such instances, the law is discursively ascribed different kinds of value, somewhat loaded with nostalgia and romanticism. In the latest coalition agreement, of October 2016, it is claimed that sustainability, by way of the Thule law, is a Greenlandic origination. Specifically to hunting the agreement states:

The principle of sustainability originates in Greenland; the first provisions about sustainable use of game (*fangstdyr*) were formulated in the Thule laws, and these principles must be claimed and glorified. (Kielsen et al. 2016, p. 14; translated from Danish by authors)

This discursive practice attaches cultural and symbolic values to hunting and hunters in Avanersuaq, and hence places the Thule area and its people centrally in Greenlandic national identity. Whether or not the sustainability principle, indeed, originates in Greenland, is not our judgement to make, but the paragraphs about the Thule law referred to in the modern coalition agreement *do* establish principles for protection of some species. The original Thule law takes as its premise that living resources are not infinite:

Every free hunter and trapper may acquire food and fur by hunting and trapping for himself and his family. However, the wildlife (*Vildtet*) is no longer present in unlimited quantities. All over the world, free peoples have, therefore, decided that wildlife must be protected during breeding season; otherwise, each year will bring less and less wildlife. In our region, the species to be protected from depletion are eider ducks, foxes, and walrus, and every free hunter should happily agree to such protection. Otherwise, these animals will be depleted once our children reach adulthood. (Kap York Stationen Thules Love af 7. juni 1929 printed 1947: 98, translated from Danish by authors)

The proposition that wildlife should be managed and protected for the good of future human generations, in a way that balances human development with that of ecosystems, is echoed by those in contemporary definitions of sustainable use of ecosystems (Brundtland et al. 1987), including in the current management goals of Greenland.

In terms of walrus hunting, the Thule law states walrus must be harpooned before shot. If a hunter broke this rule under the Thule law, there were penalties. Today, hunters in Avanersuaq sometimes emphasise the Thule law as the first time in history that restrictions were imposed on their hunting. In other situations, they emphasise the law as one they still proudly follow. When hunters still (nostalgically) accredit the law legitimacy, it is probably due to the level of self-determination it represents. Hunters were represented in the organisation that was making laws and decisions about all aspects of life in the little community. In addition, although hunters are formally heard in processes of decision-making today, the resulting regulations rarely include hunter opinions on population abundance, or the implicit notions of sustainability that guide them when hunting.

When hunters are dissatisfied with the quotas and regulation related to walrus, it is owed, in part, to the fact that regulation is perceived to intrude on local self-determination and the knowledge hunters have of wildlife. It is also owed to the economic situation in Avanersuaq where there is a little investment and limited possibilities for income. Dog sledge tourism is one of the few ways hunters maintain a monetary income, making the hunters’ procurement of walrus meat especially important to keep their dogs in good health. Inughuit livelihoods unfold far away from the places where sustainability principles have been formulated and adopted in the Rio Declaration (which was signed by Greenland in 1992). In contrast, the Thule law was formulated for the particular area where it was applied. It entered the hunter-community network as the first formal restriction on hunting in Avanersuaq. Although the law has long been legally obsolete, it is still present in the hunter-community network, memorised as a regulation decided by hunters along with administrators. Second, the law enters the contemporary politics-management network when politicians in Nuuk point at the Thule law to glorify national traditional hunting practices as sustainable.

## Conclusion: Walrus and sustainability contested

When considering walrus within the three networks: hunter-community, knowledge-biology, and politics-management different actors become visible, some located far beyond the geographical limits of Avanersuaq and Smith Sound. Decisions about walrus are made far away from the animals, and the people who hunt them, however the effects of these decisions can directly or indirectly impact hunters in Avanersuaq.

Sustainability appears with different implications at different points in these extensive networks. In at least one sense, the concern of the Greenland Government about sustainable walrus hunting is of international scope. For export of walrus tusks it is required that each ivory item is supported by a Non-Detrimental Finding (NDF) issued by the Greenlandic scientific CITES authority. The Greenland Institute of Natural Resources (GINR) issues such statements if the quotas of all three Greenlandic stocks of walrus are following the catch levels recommended by NAMMCO. In 2016, however, GINR declared hunting of walrus negative in terms of hunting being sustainable, since the catch levels in Qaanaaq superseded the NAMMCO recommendations (GINR 2016). This was reversed in 2017 where quotas followed the advice. In this respect, the international concern about wildlife exploitation has impacted the communities living in Avanersuaq. Sustainability, here, becomes an operational criterion, measured through biologist procedures.

The pertinent question remains whether or not sustainable resource utilisation is relevant and applicable as a concept in Avanersuaq. The walrus with its local importance and international oversight is an obvious case for exploring this question. In this article, we have unpacked the different meanings and implications of sustainability by tracing the walrus through different networks, and we have shown that a straightforward answer to the question is not possible without doing violence to complexity. We have shown how the walrus comes to sustain different values as it moves through different networks: hunter-community, knowledge-biology, and politics-management. The walrus is transformed when circulating through these networks, thereby becoming a resource in more than one sense. In the Avanersuaq hunter-community network, the walrus sustains particular households, providing meat for dogs and humans, and materials to use or craft for goods that can be sold. The walrus also sustains hunting as a livelihood, and cultural specific modes of relating to the landscape and animals. We have shown that sustainable resource utilisation is not a concept integral to hunting, although notions of respect towards animals and hunting grounds implicitly have been practiced and passed on through generations. This network is, however, not a closed system, and seen from this network, the concept is not per se relevant and applicable, because walruses are not considered in danger of depletion. Changes in walrus availability (due to hunting restrictions, changing ice conditions or changes in the walrus population) are only one factor putting pressure on social and cultural aspects of livelihoods in Avanersuaq. Other changes are happening with globalisation, climate change, and introduction of mineral extraction in hunting areas. In this sense, international regulation of wildlife utilisation affects hunters in Avanersuaq, and therefore, sustainable resource utilisation becomes a relevant and applicable concept.

In the knowledge-biology network, the walrus sustains scientific practices and ways of knowing the environment through singling out species that can be studied and monitored separately. From a biological perspective, sustainable resource utilisation is a relevant concept in Avanersuaq, because the heavily subsidised maritime hunting culture has undergone significant changes in hunting effort, and second, because they are subject to global observation of wildlife exploitation. Biologists work actively with the concept of sustainable resource utilisation, providing information on past and present utilisation, and generating models on future abundance.

Differences in biologists’ and hunters’ ways of understanding and assessing the amounts of animals available are primarily rooted in different ways of understanding and knowing the environment; one independent of the resource utilisation based on observational techniques (also called ‘western natural science’), and the other intermingled with the resource utilisation, rooted in local livelihoods and generations of living in and of the landscape and ecosystem (often also framed as ‘indigenous knowledge’ (Freeman 2011)). As we have shown, the walrus networks and the different ways of knowing are not isolated, but connect and overlap.

In the politics-management network, the walrus and walrus hunting sustain national Greenlandic identity. Greenlandic decision-makers (politicians) are, however, also concerned about creating an economy that can sustain a path towards political independence. Economic growth and sustainability are dependent on the total sum of natural resources and on international markets willing to import products, knowing that no overhunting on endangered or beloved species is taking place. In this network, sustainable resource utilisation is relevant as a concept, since it serves to monitor and prove that international standards of wildlife use and management are met.

Core disagreements between hunters and decision-makers include the kind of information used for developing regulation, and who should be at the table to make decisions about wildlife harvest.

We conclude that sustainable resource utilisation is a relevant concept in Avanersuaq and beyond, since as concept and phenomenon it has actual effects on walrus-human relations. However, as we have shown, the term sustainable resource utilisation is also problematic as a generalised concept, and a standardised tool of management, because it ignores manifold levels of human and societal values and modes of engaging with a local environment. All the while complicated biologist efforts work towards standard notions of sustainable catch levels and sustainable resource utilisation, these notions are destabilised and meanings multiply as the walrus enters other networks. Neither sustainability nor resource is terms that carry the same meaning and value as they are applied and travel from document, legislation, practices, declarations, statement, or assessments.

The walrus population in Smith Sound sustains various interests. The networks and actors around the walrus make the walrus take different forms. It is our argument that the manifold versions of walrus, and values tied to it, must be considered when assessing sustainability and sustainable resource utilisation. This means that definitions of sustainable resource utilisation must continuously be assessed and evaluated in relation to particular constellations of human livelihoods and animal habitats. Furthermore, when applied in management, the concept should be redefined to fit particular ecosystems, and specific kinds of resource use, including diverging modes (those of hunters and biologists) of conceptualising human–environment relations as well as different ways of knowing and valuing the environment. Modern forms of management should find modes of integrating multiple and complex meanings and values into decision-making procedures.

## Footnotes

1 While writing this paper, a revision of the Greenlandic Government’s executive order on protection and hunting of walrus was under revision. The revised version as well as the one in force from 2006 (*Hjemmestyrets bekendtgørelse nr. 20 af 27. oktober 2006 om beskyttelse og fangst af hvalros)* both require harpooning before shooting.

2 Atlantic walruses, apart from those in Smith Sound, are divided into seven different stocks, of which two more occur in Greenland: the Hudson Bay–Davis Strait stock in West Greenland, and the East Greenland Stock (Wiig et al. 2014).

3 See ‘Hjemmestyrets Bekendtgørelse nr. 20 af 27. oktober 2006 om beskyttelse og fangst af hvalros’.

4 When writing this article, DKK 100 equaled USD 14,33.

5 For more statements by hunters in Avanersuaq about the management of walrus and the impact of quotas, see Born et al. (2017).

6 *Piniarneq* is the existing catch reporting system in Greenland. It was introduced in 1993.

7 Articulations of what is often denominated animism are more common in literature from Canada, and are less articulated in Greenland. This absence in the current literature does not mean that such practices and notions are completely absent in Greenland. Rather, it is an effect of less anthropologists having worked in and described Greenland Inuit than Canadian, and differences in particular historical and colonial relations in the two countries, which render these beliefs discernible and important in political and scientific spheres in different ways.
